# Supplementary material for: Are carbon emissions trading and green financial instruments synergistic? -Comprehensive quantitative research based on content analysis
Source: PLoS One. 2024 Mar 7;19(3):e0298601. doi: 10.1371/journal.pone.0298601 (PMC10919601; doi:10.1371/journal.pone.0298601)
Supplement: S1 Data — (ZIP) [file pone.0298601.s001.zip › Data/地级市Z_F工作报告.zip!/║┌┴·╜¡/╣■╢√▒⌡╩╨/└┤╫╘╣1⁄2╓┌║┼í╛┬φ┐╦╩2╛▌═°í┐_2006.docx]

各位代表：

　　现在，我代表市人民政府向大会作工作报告，请予审议，并请市政协各位委员提出意见。

　　一、2005年及“十五”时期工作回顾

　　“十五”时期，我们坚持以邓小平理论和“三个代表”重要思想为指导，树立和落实科学发展观，坚决贯彻党中央、国务院和省委、省政府的方针政策，紧紧把握市委“努力快发展，全面建小康，振兴哈尔滨”的工作主题，全力推进“五个基地、一个中心、一座名城”建设，经济社会步入快速健康发展轨道。2005年，实现生产总值1830.4亿元，年均增长13%。财政一般预算收入165.5亿元，年均增长14.1%，其中地方财政一般预算收入98亿元，年均增长12.7%。完成固定资产投资639亿元，年均增长20.3%。社会消费品零售总额788.1亿元，年均增长11.6%。

　　快发展、大发展的产业基础初步形成。三次产业结构由2000年的19.3∶31.2∶49.5调整为16.4∶35.3∶48.3。工业主导作用进一步增强。2005年完成工业增加值490.9亿元，年均增长15.7%，是改革开放以来增长最快的时期。工业增长质量显著提高，经济效益综合指数由2000年的74.2提高到128.4。优势产业逐步做大趋强，机械、食品、医药产业实现增加值227.8亿元，占规模以上工业的65%。电站、哈航、哈啤、哈药等骨干企业综合实力居全国同行业领先地位。县域经济快速发展，经济结构得到优化。粮食产量达到92.5亿公斤，创历史新高。畜牧业发展势头强劲，增加值占农林牧渔业的比重达到51.7%。获得认证的无公害、绿色和有机食品种植面积达到1785万亩。农业产业化经营不断发展，带动农户和拉动农民增收的水平有较大提高。第三产业实现增加值884.5亿元，年均增长12.9%。商业格局日趋完善，商业业态实现多元化。旅游业由小到大，成为全国主要旅游目的地城市之一，冰雪旅游发展成国际化知名品牌。2005年旅游业总收入122.6亿元，年均增长22.2%。物流、金融、会展、交通、房地产、信息咨询等服务业稳步发展。

　　城市发展空间实现历史性扩展。经过行政区划调整，城区面积扩大到4272平方公里，是原来的2.6倍。编制完成新一轮《哈尔滨市城市总体规划》，半径为100公里的都市圈建设开始启动。实施“开发江北，两岸繁荣”战略，对松北区、呼兰区进行整体开发，启动哈西、哈东、顾乡、群力、新香坊、平房工业新城等区域性调整改造，松花江南北两岸联动发展的态势初步形成。实施《哈大齐工业走廊哈尔滨段规划》，完成总投资43.8亿元，161户企业签订入区协议，其中65户企业开工建设。

　　城市功能不断完善，特色更加突出。树立科学的城市建设和管理理念，由建设“水泥城市”向建设生态型园林城市转变，由单纯城市功能建设向功能建设与文化延展并重转变。建立有效的城市建设和管理机制，实行市区联动、区企联动、重心下移，推动城市面貌和环境质量发生重大变化。五年完成城市建设投资493亿元，是“九五”时期的2.2倍。一批具有深远影响的基础设施项目取得重大进展。新建4座大型桥梁，建成二环快速干道，拓宽改造141条道路，建设通乡通村公路1595公里。新建、改造供水管网250公里。磨盘山水库供水一期工程实现下闸蓄水。实施道里集中供热、西南部集中供热等13项工程，新增集中供热面积4683万平方米。完成沿江生活污水截流、文昌污水处理厂二期、太平污水处理厂等工程，城市污水日处理能力达到65万立方米。建成3座大型垃圾无害化处理场，城市生活垃圾无害化处理能力达到85%。新增供气管网334.4公里，燃气普及率达到95%。削减水、气污染物2万吨，全年空气质量良好天数达到301天。城市文化品位不断提升。建设改造中央大街、果戈里大街、印度风情街、高丽一条街、三大动力路、哈药路等文化街区，实施太阳岛综合整治、沿江风景长廊改造、北方森林动物园等工程，完成哈尔滨关道复建和犹太会堂、清真寺等综合改造工程，辟建市民广场、俄罗斯河园、巴黎广场、维也纳音乐广场等50个区域性广场。加大环境整治和城管综合执法力度，城市管理水平不断提高。城区新增绿地1962公顷，植树428万株，绿化覆盖率28.35%。铺装改造背街巷道1178条，建成无棚厦生活片区300个、达标景观庭院1118个，安装500条无照明街路路灯。实施了火车站广场、博物馆地区和铁路沿线等重点区域环境整治。完成造林304.8万亩，全市森林覆盖率达到44%。

　　各项改革取得重大进展。国有企业改革步伐不断加快，2003年以来完成国有企业改制1403户，引入投资26.5亿元，转让国有资产收益27.8亿元，其中2005年完成1113户。哈啤、哈药、哈水泥厂、哈一百等20户企业实现战略性重组。配套改革进展顺利，分离移交71所市属企办学校和19所企办医院。农村改革不断深化，撤乡并村和税费改革基本完成，减轻农民负担工作实现历史性突破。开展农村信用社改革试点工作。事业单位改革稳步推进，清除自行设立机构134个、编制8575个，撤销、合并事业单位156个，精简事业编制3567个。

　　对外开放步伐加快。2005年海关进出口总额27.1亿美元，年均增长17.5%。出口产品结构不断升级，机电、高新产品出口增势强劲，具有自主知识产权的微型车出口量居全国同行业第一。推进对俄经贸科技合作战略升级，对俄贸易稳步增长，科技合作成果显著。“哈洽会”升级为国家级展会，确立了中国对俄经贸第一展会的地位。通过“香港活动周”、“韩国周”、“东盟博览会”等活动，搭建了对香港和东南亚国家的招商引资平台。五年实际利用外资15.1亿美元，在哈尔滨市投资的国家和地区54个，世界500强企业18家。

　　人民生活水平显著提高。2005年城镇居民家庭人均可支配收入10065元，农民人均纯收入4006元，年均增长12.3%和10.1%。居民消费结构发生显著变化，住房、教育、汽车、旅游渐成消费热点。城市人均住房使用面积达到16.7平方米，农村居民住房砖瓦化率达到77.7%。五年安置城镇社会新就业人员57万人，城镇登记失业率控制在5%以下，32.8万国有企业下岗职工基本生活保障实现向失业保险“并轨”，城镇职工养老保险、失业保险和医疗保险覆盖面分别达到84%、88%和65.5%，确保40万离退休人员养老金按时足额发放，11.4万人领取失业保险金，30.5万人次享受住院医疗保险待遇。加大对困难群众救助力度，142.6万人次享受城市最低生活保障，支付低保资金7.8亿元；供暖补贴、供水补贴、教育助学、法律援助等专项救助政策进一步落实；改造危房棚户区585万平方米，为特困家庭解决廉租住房1127套，完成人均住房6平方米以下廉租户的住房解困工作。

　　各项社会事业协调发展，精神文明建设全面推进。科技事业快速发展，综合科技实力不断增强，获得全国科技进步先进城市称号。五年共取得科技成果4733项，申请专利12607件，高新技术产业产值年均增长32.2%。教育改革不断深入，布局和结构得到优化，教育质量进一步提高，教育技术信息化建设居全国领先地位。文化事业异彩纷呈，群众文化活动不断创新。卫生事业发展步伐加快，完成公共医疗卫生体系建设项目27个，农村新型合作医疗试点逐步扩大。人口自然增长率控制在4.3‰以内。世界大学生冬季运动会筹备工作全面展开，竞技体育取得新成绩，全民健身活动蓬勃开展，安装健身路径1050件（套）。深入开展群众性精神文明创建活动，弘扬“开放包容、时尚活力、诚信敬业、和谐奋进”的城市精神，获得全国文明创建工作先进城市和双拥模范城“五连冠”称号。健全信访工作机制，化解人民内部矛盾。继续打击“法轮功”等邪教组织。深入开展创建“平安哈尔滨”活动，刑事案件综合发案率不断下降。

　　行政能力不断增强。坚持依法行政、转变职能、改进作风和反腐倡廉。自觉接受人大依法监督和政协民主监督，办复人大代表建议2488件、政协委员提案4449件，办复率100%。加强法治政府建设，起草审核27件地方性法规草案，制定74件规章和102件规范性文件。深化行政审批制度改革，审批事项由1191项减少到172项。组建市行政服务中心，44个部门入驻中心集中审批，实行“一站式”办理、“一条龙”服务、“一个窗口”收费。推进行政效能建设，从严整治行政“不作为”和“乱作为”，提高了行政效率和公共服务质量。加快推进政务公开，建立并落实重大决策咨询、信息公开等制度，推进电子政务，“中国哈尔滨”网站在国家测评中排名提升到第10位。坚持“课题制”的方法，有效解决了经济社会发展中的一些热点、难点问题。五年来，我们经受住了“非典”疫情等重大突发性公共危机的严峻考验，特别是面对突如其来的松花江水污染事件，我们紧紧依靠党中央、国务院、省委、省政府和市委的领导，依靠广大人民群众的支持，快速反应，沉着应对，果断决策，科学处理，取得了决定性胜利。

　　各位代表，过去的五年，是哈尔滨市改革开放向纵深推进的五年，是城市面貌显著变化的五年，是人民群众生活水平显著提高的五年，是弘扬城市精神、创建文明城市取得明显成效的五年。五年来，哈尔滨市主要经济社会指标持续以两位数的速度增长，综合实力连续两年跻身全国城市前10位。这些成绩的取得，得益于党中央、国务院、省委、省政府和市委的正确领导，得益于市人大、市政协的大力支持，得益于中、省直各部门的关心和帮助，得益于驻哈人民解放军、武警部队的大力支持和海外同胞、国际友人的广泛参与，凝聚着970万哈尔滨人民的辛劳和智慧。在此，我代表哈尔滨市人民政府，向所有参与、支持和关心哈尔滨发展的同志们、朋友们表示衷心感谢并致以崇高敬意！

　　五年的工作实践，使我们加深了对科学发展观的认识，增强了运用科学发展观推进经济社会发展的自觉性。我们体会到，在推进经济社会发展上，不仅要扎实做好具体工作，更要站在战略高度，从可持续发展的视角，谋划哈尔滨的整体振兴；在把握发展机遇上，必须与国家和省的政策形成互动，努力做好“结合”的文章；在推进和谐社会建设上，必须坚持以人为本、与人为善，把关注民生、维护民利、排解民忧作为政府工作的出发点和落脚点；在政府工作中，必须不断解放思想、创新思路，在重点、难点问题上攻坚克难。

　　哈尔滨市经济社会发展仍然存在一些根本性、长期性和深层次问题。一是经济结构不尽合理，主导产业优势不明显；二是体制性矛盾仍然比较突出，改革任务还很艰巨，非公有制经济有待进一步发展；三是农业和农村经济持续发展的基础比较薄弱，农民增收难度依然很大；四是自主创新能力不强，经济增长方式没有实现根本性转变；五是外来投资规模小，经济外向度偏低；六是社会矛盾和问题还比较多，就业压力大，社会保障能力弱，“看病难”、“上学难”等问题还没有从根本上解决；七是松花江水污染影响需要综合治理、逐步根除；八是政府职能转变还没有到位，公务员队伍的整体素质有待进一步提高，经济发展环境还需继续改善。我们将高度重视这些矛盾和问题，采取有效措施，认真加以解决。

　　二、“十一五”时期主要任务和2006年工作

　　“十一五”时期是全面振兴老工业基地的关键时期，也是全面建设小康社会的重要时期，我们面临着极为有利的发展条件。经济日益全球化，特别是制造业向中国转移，将进一步助推哈尔滨市外向型经济发展和产业优势的发挥；中俄两国战略协作伙伴关系不断发展，有利于哈尔滨市进一步发挥区位优势，推动对俄经贸科技合作战略升级；国家加大对老工业基地和粮食主产区扶持力度，省委、省政府支持哈尔滨市“加快发展、当好龙头”，推进哈大齐工业走廊建设，使哈尔滨市面临更优越的政策环境；已经构建的产业基础和城市格局，为快发展、大发展提供了更大的空间和可能；2009年在哈尔滨市举办的第24届世界大学生冬季运动会，对带动经济社会发展，提升城市形象将发挥重要作用；在抗击突发性公共危机事件过程中，全市上下体现出顾全大局、团结互助、无私奉献、敢于胜利的精神，将成为推进哈尔滨发展的强大动力。我们必须抓住机遇，乘势而上，以邓小平理论和“三个代表”重要思想为指导，贯彻党的十六大和十六届五中全会精神，以科学发展观统领经济社会发展全局，推进全面小康社会和社会主义和谐社会建设。走新型工业化道路，转变经济增长方式，全面实现老工业基地振兴。统筹城乡经济社会发展，推进社会主义新农村建设。整合以文化为领航、科技为先导、教育为基础、人才为根本的自主创新能力，推进创新型城市建设。坚持以人为本、民生优先的发展取向，完善城市功能，加快社会发展，优化空间布局，强化资源节约，加强环境保护。建设国家重要的机械制造业、高新技术产业、绿色食品、医药工业和对俄经贸科技合作基地，东北亚重要的经贸中心和世界冰雪旅游名城，使哈尔滨成为适宜创业、适宜人居、适宜人的全面发展的现代文明城市。

　　“十一五”时期，哈尔滨市经济社会发展目标是：经济持续快速协调健康发展，全市生产总值预期年均增长12%以上。经济增长方式进一步转变，产业层次和竞争力明显提升，形成一批拥有自主知识产权和知名品牌、国际竞争力较强的优势企业。资源利用效率显著提高，单位生产总值能源消耗比“十五”期末降低20%，城乡环境质量有较大改善。城市空间结构和功能布局更趋优化，城市建设和管理水平不断提高，城市环境更加优美宜人。社会事业全面进步，城镇就业岗位持续增加，社会保障和救助体系比较健全，城乡居民收入水平和生活质量普遍提高，居住、交通、教育、文化和卫生等方面的条件有较大改善。民主法制建设和精神文明建设不断强化，社会整体文明程度和市民素质不断提升，社会治安和安全生产状况进一步好转，构建和谐社会取得新进步。

　　实现上述目标，必须全面贯彻落实科学发展观，突出重点，在以下几个方面强力推进。

　　———保持经济社会又快又好发展。紧紧抓住各种发展机遇，充分利用一切有利条件，引导好、发挥好各方面积极性，在不断提高增长质量和效益的前提下，保持较快发展势头。

　　———不断深化改革，扩大开放。坚持社会主义市场经济的改革方向，完善现代企业制度和产权制度。切实转变政府职能，最大限度地发挥市场在资源配置中的基础性作用，提高资源配置效率。不断提高对外开放水平，积极参与国际合作与分工，增强在扩大开放条件下促进发展的能力。

　　———加快经济增长方式转变。加快国民经济和社会信息化，坚定不移地走新型工业化道路。发展循环经济，保护生态环境，高效利用资源。坚持节约发展、清洁发展、协调发展、安全发展，增强发展的可持续性。

　　———着力提高自主创新能力。深入实施科教兴市、人才强市战略，把增强自主创新能力作为调整产业结构、转变增长方式的中心环节，提高原始创新、集成创新和引进消化吸收再创新能力，建设创新型城市。

　　———促进城乡区域协调发展。着眼于改变城乡二元结构，实行工业反哺农业、城市支持农村，推进社会主义新农村建设。加快都市圈建设和城乡一体化进程，形成城乡优势互补、良性互动的区域协调发展机制。

　　———加快社会主义和谐社会建设。从解决关系人民群众切身利益的现实问题入手，更加注重经济社会协调发展，加快发展社会事业，促进人的全面发展；更加注重社会公平，使人民群众共享改革发展成果；更加注重民主法制建设，正确处理改革发展稳定的关系，保持社会安定团结。

　　2006年是实施“十一五”规划的第一年。按照市委十一届四次全会精神，2006年哈尔滨市经济社会发展主要预期目标是：地区生产总值增长13%，全社会固定资产投资增长25%，地方财政一般预算收入增长9%，社会消费品零售总额增长13%左右，市属进出口总额增长20%，实际直接利用外资增长20%，城市居民人均可支配收入增长9%，农村居民人均纯收入增长6%,居民消费价格总水平涨幅控制在3%以内,城镇登记失业率控制在4.6%以内，万元GDP综合能耗降低4%。

　　(一)继续调整和优化产业结构，强化工业经济主导作用

　　围绕振兴老工业基地，优化产业结构，提高工业经济整体竞争力。工业增加值增长16.5%。

　　调整产业布局，提高产业集聚能力。加快发展机械制造、高新技术、食品、医药4大优势产业，延伸产业链。机械制造业重点发展发电设备、汽车、航空航天及配套等5个产业链。食品工业重点发展乳制品、啤酒、肉类等5个产业链。医药工业重点发展抗生素、化学医药、现代中药、疫苗及兽药等系列产品。高新技术产业重点发展光机电一体化、电子信息、生物工程、新材料、高效节能与环保、现代农业等产业和产品。加快发展化工产业和木材加工业。

　　推进大项目建设，提高产业能级。加快实施具有自主知识产权、市场前景好、能突破关键技术瓶颈的重大项目，提高投资效益。重点抓好超大规格铝板带材、4G9发动机等50个投资超亿元的项目，加快实施锅炉膜式壁、齿轮测量中心等190个投资超千万元项目。工业投资额增长20%以上。

　　科学规划和发展工业园区。优化调整工业园区，形成具有自身特色的产业板块。加快发展利民经济技术开发区、双城经济技术开发区和宾西经济开发区等园区，培育25户龙头企业，加快实施年产3万吨谷氨酸等30个大项目。

　　加快哈大齐工业走廊哈尔滨段的规划建设。完善总体规划，注重功能和设施配套，制定和实施江北工业新区、太平空港经济区等5个启动区的控祥规划，加快基础设施建设。引进国际著名大企业、大财团等战略投资者，建设对俄罗斯、韩国等产业园区。

　　（二）加快区县经济发展，推进社会主义新农村建设

　　围绕农民和区、县（市）财政双增收目标，坚持以工促农、以城带乡，推进农村工业化、农业产业化、农业科技化和农村城镇化，实现社会主义新农村建设的良好开局。

　　优化农业和农村经济结构。稳定粮食种植面积，提高粮食综合生产能力。推进标准化生产，扩大优质品种面积，粮食作物标准化面积增长20%。加快发展畜牧业，加强重大动物疫病防控，规模化养殖比重达到60%。推进绿色食品产业升级，无公害、绿色和有机食品生产面积突破2000万亩。加快发展农业产业化经营，做大做强龙头企业，市级以上龙头企业发展到52个，联结农户61万户。发展农副产品深加工，强化特色产业集聚效应。加快城乡工业协作分工体系建设，引导区县企业加入城市产业发展链条，推进产业对接。加强财源建设，大力发展重点财源项目。发展农村第三产业，开拓农村消费市场。

　　建设新型村镇。推进小康示范镇建设。实施“442”工程，支持4个县（市）争创全国百强县（市）,扶持40个区域中心镇和200个中心村加快发展。实施村镇建设新农村示范工程，建设10个示范村、30个达标村和20个试点镇。加快中小型水利工程改造和大型灌区建设，搞好龙凤山、友谊、香磨山、倭肯河等大型灌区节水改造。实施农村饮水安全工程，解决4.8万人饮水问题。推进生态修复工程建设，完成水土流失治理面积50万亩，造林30万亩，退耕还林4万亩。启动1000公里通乡通村公路建设，实现通车500公里。

　　大力发展农村公共事业。扩大公共财政覆盖农村的范围。发展农村义务教育，改善农村办学条件。强化农村卫生工作，乡村卫生服务管理一体化普及率达到80%以上，启动阿城、尚志新型农村合作医疗试点工作，规范发展农村便民医药连锁店。采取政策措施，解决失地农民养老保险问题。加强农村文化、体育设施建设，丰富农民生活。推进农村社会救助体系建设，做好灾害救助工作。

　　拓宽农民增收渠道。加强农业实用技术培训和职业技能培训，强化农民进城务工的管理、维权和服务，增强农民工就业能力。加快发展县域二、三产业，吸纳农民进入非农领域。全年培训农民工30万人，转移农村劳动力110万人，实现转移劳务收入45亿元。

　　完善农业和农村经济管理体制。全面落实土地承包政策，建立土地使用权依法流转机制。推进乡镇机构、义务教育、乡村财政管理体制改革，巩固和发展农村税费改革成果，化解乡村债务。深化农村金融体制改革，规范发展金融组织，探索发展农业保险。大力发展农村新型合作经济组织，提高农民组织化程度。

　　（三）加快发展现代服务业，优化第三产业结构

　　推进服务业市场化、品牌化和国际化，增强综合服务功能。第三产业增加值增长12.5%。

　　大力发展旅游、会展业。坚持旅游与会展相结合，强化“哈洽会”、“哈夏音乐会”、“冰雪节”三大品牌，培育夏季清凉旅游品牌，提高哈尔滨国际啤酒节、依兰漂流节、阿城金源文化节等活动的层次和水平，推进天恒山风景区、龙凤山风景区等项目建设。推动东北地区城市间合作体系建设，共同打造旅游无障碍区。全年接待旅游者人次增长18%，旅游业总收入增长19%。

　　加快发展商贸流通业。实施《哈尔滨市现代物流业发展规划》和《哈尔滨市商品市场体系（商业网点）建设规划》。引导和推进物流基础设施建设，重点发展第三方物流，加强东北四城市物流合作。支持连锁经营、特许经营、电子商务等新兴业态发展。

　　稳步发展房地产业。优化住宅供应结构，大力发展普通商品住宅，积极发展节能省地型住宅，试行经济适用住房货币补贴办法，稳定住房价格。完善征地拆迁制度、土地收购制度和国有土地使用权公开招标、拍卖、挂牌出让制度。控制住房与房地产开发建设总量，科学利用土地资源。

　　努力促进消费需求增长。培育汽车、住房、旅游等新消费热点，加快餐饮业业态创新和结构优化，鼓励支持家政、中介、技术服务等新兴服务业发展，促进消费升级转型。鼓励企业在农村发展连锁经营，搞好“万村千乡”市场建设工程，改建300家“农家店”。

　　大力发展金融保险业。加强银政联手和银企合作，加大金融对经济发展的支持力度。完善地方金融体系，改善金融环境。大力发展地方金融机构，推进社会信用体系建设，做大做强融资担保机构，筹建哈大齐工业走廊融资担保公司。鼓励外资银行在哈尔滨市设立机构、开展业务。支持保险业规范健康发展，拓展农村保险市场。

　　（四）加强城市规划、建设与管理，提高城市承载发展的能力

　　加强城市规划管理。编制哈尔滨近期建设规划、公共安全体系规划，完成重点地段控详规划、迎接“大冬会”专项规划、松花江沿岸大顶子山至哈尔滨段专项规划，力争完成各县（市）全部乡镇总体规划编制任务。加强规划法规体系建设，提高规划管理水平。

　　加快都市圈建设。实施城镇化战略，加快中等卫星城市和重点小城镇建设。推进主城区功能化建设，加快呼兰、松北、哈西、哈东、顾乡、群力整体开发，搞好香坊工业新区、平房工业新城、动力新商圈、承德广场等区域性改造。

　　加快城市基础设施建设。实施“一环六线十桥百路”工程。启动三环路建设工程，实施轨道交通一号线、机场路迎宾线等6项工程，建设道外二十道街跨江桥、学府路立交桥等10座桥梁，新建改造公滨路等100条道路。加快实施城市供水、供热及污水、垃圾处理工程，推进磨盘山水库、西泉眼后备水源、水厂改造以及达连河等沿江供水工程建设，完成道里集中供热三期工程，续建西南部集中供热和热电厂扩建工程，启动哈东集中供热、平房工业区供热等工程建设，市区集中供热普及率达到61%；完成文昌污水处理三期工程，启动群力、信义沟、呼兰河、阿什河等11个污水处理工程，城市生活污水集中处理率达到60%以上。建设松北垃圾焚烧发电处理厂工程。推进群力堤防工程建设。加快城市建设市场化步伐，整合建设系统融资平台，完善城市建设项目库，吸引外资和社会资本参与大项目建设。

　　推进城市环境建设。实施兆麟街区开发、沿江风景线升级等综合整治工程，启动萧红故居扩建、文庙区域整治、俄罗斯风情园、高丽风情街扩建、黛香湖公园等工程。启动何家沟生态线建设。加快城区绿化，新增绿地700公顷，植树150万株，新建改造一批高标准绿地、公园、庭院和景观大道，达到省级园林城市标准。加强街路和居民区环境整治，改造一批早期建设的居民小区，加快市政土路、背街巷道改造及人行道铺装，建设改造一批无灯街路、水冲公厕和垃圾转运间。深入开展创建国家卫生城市和环保模范城市活动。

　　提高城市管理精细化水平。整合城市管理资源，构建以市为主导、区为主体、街道和社区为基础的城市管理新体制。推进城市管理进社区，构建数字城管新模式。加强占挖道路、市场摊区、乱贴乱画和建筑垃圾残土运输等专项治理，提高街路清扫保洁、道路维修养护、园林绿化抚育、清冰雪等管理水平。加大城市管理综合执法力度。推进供热管理体制改革，完善城市物业管理。

　　（五）深化改革，增强经济发展动力

　　基本完成国有企业改革任务。推进燃气化工总公司、建工集团及交通系统企业改制进程，做好哈轴集团、东轻公司、电缆厂等企业改制。启动国有企业厂办大集体改制工作。加快学校、医院等企办社会职能分离。开展国有资产经营预算试点，健全国有控股企业法人治理结构，依法加大对国有企业的监管力度。

　　加快发展民营经济。完善政策措施，支持非公有制骨干企业做大做强，健全非公有制企业参与国有企业改革的机制。从今年起，市级财政每年安排5000万元资金，用于提高对非公有制企业的担保能力，安排1000万元设立市中小企业发展专项资金。

　　深化财政体制改革。推进以国库集中支付、政府采购和收支两条线为重点的财政管理制度改革，开展财政支出绩效评价试点，制定科学规范的转移支付办法和有效的监管制度，创新财政管理制度和理财方式，完善财政资金分配运行机制，提高财政资金使用效益。加强财政刚性预算，提高预算透明度和约束力。启动住房货币化分配。

　　加快事业单位改革。做好综合配套改革的各项后期管理工作，全面实行“联动管理”。推进财政补助事业单位分配制度改革试点，推行后勤服务社会化和社会保险制度改革。

　　（六）扩大对外开放，提高经济发展外向度

　　以对俄经贸科技合作战略升级为重点，加快发展对外经济贸易。以“俄罗斯年”活动为契机，加强对俄经贸合作，加快对俄合作产业园区、国际贸易加工区、东北亚经贸科技合作区、对俄出口加工区等基地建设，完善对俄贸易大通道。加快对俄科技合作产业园区建设，组建对俄工业研究院等对俄科技合作中心，推进轻型飞机复合材料等60个重大项目实施。大力开拓国际市场，培育外贸骨干企业，实施品牌战略，优化出口产品结构，转变外贸增长方式。办好第17届“哈洽会”等大型经贸活动。

　　加大招商引资力度，实现利用外资的重大突破。围绕国有企业改革、产业结构调整、城市基础设施建设，积极开展招商引资活动。继续推进产业招商、园区招商、会展招商、网络招商等招商形式。建立健全“招商代表制”，大力推行委托招商，借助中介组织的招商网络和跨国公司的影响，扩大引资规模。支持开发区二次创业。

　　加强国内经济合作，提升区域合作发展水平。抓住发达地区产业转移的机遇，开展多形式、多层次、多领域的联合与协作，在资源开发、科研教育、产业整合等方面实现互动互补。加强与东北其他城市的交流与合作，推动区域间市场互通、产业互补、设施共建、信息共享。

　　（七）提高自主创新能力，加快建设创新型城市

　　组织制定哈尔滨市建设创新型城市规划，深入实施“科教兴市”、“人才强市”战略，坚持自主创新、重点跨越、支撑发展、引领未来的方针，深化科技教育体制改革，力争在重点领域实现突破。高新技术产值增长20%，专利申请量3000件。

　　完善科技创新体系。强化企业主体地位，建设铝镁新材料、电站成套设备、支线飞机等国家级高新技术基地，加快工程技术研究中心、国际技术转移服务中心和技术产权交易中心建设。鼓励企业增加研发投入，促进“产学研、政金介”合作，加强原始创新、集成创新和引进消化吸收基础上的再创新，推进企业技术难题的联合攻关和新技术推广。加大对区、县（市）重大科技产业化项目支持力度。

　　加强科技创新环境建设。完善政策措施，健全支持自主创新机制。整合科技资源，搭建信息交流、投融资、成果交易和科技服务平台，完善创业风险投资机制，建设装备制造业、电子信息产业、生物医药、新材料、食品安全和汽车电子6大产业联合体，提高科技成果转化率。加强知识产权的创造、管理、运用和保护工作。

　　努力提高教育水平。围绕培养创新型人才，提高高等教育质量，加强职业教育，促进民办教育有序发展。加快普及十二年教育。加强薄弱学校建设，重点改造一批薄弱学校。大力扶持农村教育发展，加强农村师资力量，加快农村学校基础设施建设和危房改造，推进农村寄宿制学校建设。

　　拓展培养、吸引、使用人才新领域。加强高级研发人才、复合型人才和技能型人才队伍建设，大力开发农村人才和高技能人才资源，积极吸引国内外高层次人才。健全人才市场体系，加强人才信息网络建设。设立“市长人才特别奖”，重奖有突出贡献的各类人才。

　　（八）推进资源节约型、环境友好型社会建设，增强可持续发展能力

　　加强能源资源节约和综合利用。加快建立以企业为主体的节能技术创新体系，加强资源节约与综合利用技术改造，推进新产品、新技术、新设备的开发、推广和应用。实施节约生产，鼓励企业生产和使用节能产品，应用先进技术。强化节约意识，大力倡导节能、节水、节材、节地，逐步形成健康文明、节约资源的消费模式。

　　大力发展循环经济。实施节能、节水示范工程和固体废物综合利用示范工程，探索“资源—产品—废弃物—再生资源”的发展模式。培育一批循环经济示范项目。制定并实施循环经济推进计划，在生产环节，严格排放强度准入，强化废物循环利用；在消费环节，实行环境标识、环境认证和政府绿色采购制度。推行建筑节能，发展绿色建筑。强化污水再生利用和垃圾处理与资源化回收，建设节水型城市。

　　加强环境保护和生态建设。制定松花江哈尔滨段水污染综合治理规划，加强对松花江水体的监测、评估和保护，推进市区生态廊道、城市生态景观、草原湿地恢复保护等生态功能区建设和管理。强化建设项目和危险化学品生产环境管理，限期治理超标排放的废水、废气，严格控制排放总量。开展生态示范县（市）、环境保护模范城区、环境友好企业和绿色社区、绿色学校等创建活动。启动“农村小康环保行动计划”，创建一批环境优美乡镇和生态示范村，着力解决养殖、土壤、饮用水安全等农村和农业环境污染问题。

　　（九）坚持“文化立市”，彰显城市个性魅力

　　整合文化资源，突出城市文化特色。以发展先进文化为主线，挖掘以音乐文化、冰雪文化、建筑文化、金源文化、异域文化、工业文化为主要内容的城市文化资源，吸收国内外现代文化成果，建设开放创新、和谐高雅的城市文化。加强历史文化名城保护工作。加大对文化事业的投入，加快公益性和基层文化设施建设，完善公共文化服务体系。高质量办好“哈夏音乐会”等大型文化活动。

　　发展文化产业。完善文化产业政策，以推进市场化和产业化为重点，加快文化体制和机制创新，提高产业竞争力。优化文化产业结构，规划建设一批产业基地，壮大企业群体。抓好精品工程，增强品牌意识，创作一批富有地方特色、反映时代精神的文化精品。加强新闻出版工作，强化文化市场管理和培育，加强知识产权保护。积极引导社会资本和外资投入文化产业，鼓励文化企业开展国际合作。

　　扎实推进社会主义精神文明建设，不断提高市民整体素质。开展形式多样的群众文化活动，丰富乡村文化、企业文化、校园文化、社区文化内容，满足人民群众多层次、多样化的文化需求，提高市民的文化素养、文明素质及精神风貌。以“弘扬哈尔滨精神、创建文明城市”为主线，创新群众性精神文明创建活动，深入开展整改“十大陋习”、弘扬“十大美德”等活动。深化未成年人思想道德建设。加强文明特色社区和文明村镇建设，推动文明单位、文明行业创建工作。

　　（十）坚持民生优先，促进社会和谐稳定

　　努力扩大就业再就业。大力发展民营企业、劳动密集型制造业、第三产业和社区服务业，增加就业岗位。认真落实《国务院关于进一步加强就业再就业工作的通知》精神，延伸再就业扶持政策。对“4050”人员、“零就业家庭”成员等困难群体，实施就业援助。加强劳动力市场建设，完善公共就业服务体系。建设职业技能培训基地，加强就业和创业技能培训，逐步建立促进就业的联动机制。搞好下岗失业人员、高校毕业生、城镇新增劳动力、农村劳动力以及退役军人的就业和安置工作。全年安置城镇就业再就业10万人。

　　完善社会保障体系。加快城镇职工基本养老保险制度改革，新增基本养老保险7万人。依法扩大失业保险覆盖范围，新增失业保险参保人数10万人。扩大医疗保险覆盖面，新增参保人员12万人。城镇参保人员基本养老保险、失业保险、医疗保险覆盖率分别达到86%、88%和68%。加强工伤保险工作，新增工伤保险参保人员13万人。多渠道筹集资金，确保社会保险待遇兑付。

　　做好扶贫济困工作。实施扶助困难群众专项行动计划，提高低保人员医疗救助水平、特殊低保对象基本生活标准和敬老院“五保”老人救助标准。发展社会福利事业，健全优抚保障机制。完善市、区、街道、社区四级捐助服务网络，规范“慈善超市”运营。设立专项资金，对特困残疾人实行专项资助。筹集教育助学金1650万元，资助贫困学生6.2万人。加快低价位商品房开发，改造危棚房50万平方米，抓好廉租住房工作。深入开展“一帮一”、“结对子”、“送温暖”等活动。进一步加强法律援助工作。

　　努力营造安全稳定的生产生活环境。坚持“安全第一，预防为主”的方针，狠抓安全生产，加强长效机制建设，坚决防止重特大事故的发生。加强交通安全监管，减少交通事故。推进防灾减灾体系建设，提高预测预报、应急救援和善后处理能力。加大价格、质量等市场监管力度，维护市场经济秩序。正确处理人民内部矛盾，及时解决人民群众反映的问题。推进社会预防犯罪工作体系建设，从源头上控制和减少犯罪。加强社会治安综合治理，搞好防控体系建设。依法严厉打击各种犯罪活动，重视和加强反恐斗争，坚决打击境内外敌对势力、敌对分子和“法轮功”等邪教组织的渗透破坏活动，积极稳妥地处置群体性事件。

　　提高人民群众健康水平。加强重大传染病防控工作，加大公共卫生突发事件应急能力建设。加大食品药品市场监管和医疗市场整顿力度，确保人民群众饮食和用药安全，逐步解决群众“看病难”、“看病贵”等问题。推进“大冬会”各项筹备工作，新建市滑冰馆，改造市冰球馆和训练馆，翻建市人民体育场。深入实施全民健身工程，安装健身路径1200件（套），建设市级体育示范社区20个。

　　加强社区建设。投入800万元专项资金，建设69个社区办公用房和公益型服务设施。加快社区卫生服务体系建设，将符合条件的卫生机构纳入城镇职工基本医疗保险定点机构。加强文化建设，强化环境综合治理，加快信息化、志愿者队伍和自治组织建设，大力开展社区建设示范活动。

　　贯彻落实计划生育基本国策，大力实施妇女儿童发展规划，稳定低生育水平，人口自然增长率控制在5‰以内。继续做好统计、外事侨务、对台、民族宗教、老龄、残疾人事业、武装、人防、气象、档案、地方志等工作。

　　三、加强自身建设，提高行政能力

　　着力提高公共服务能力。进一步转变政府职能，把工作着力点放在加强经济调节、创造良好环境上来，放在促进社会事业发展和建设和谐社会上来。强化公共服务和社会管理职能，健全公共应急体系，提高保障公共安全和处置突发事件能力。推进行政审批制度改革，减少审批事项，规范审批行为。完善行政服务中心功能，强化行政许可网上审批功能，提高服务效能，降低行政成本。完善网络基础设施建设，强化信息资源开发、整合和共享。

　　深入推进依法行政。认真贯彻人大及其常委会决议，完善工作报告制度，自觉接受人大依法监督、政协民主监督和人民群众的社会监督，做好人大代表建议、批评、意见和政协提案的办理工作。广泛听取各民主党派、人民团体的意见和建议，支持工会、共青团、妇联等群团组织开展工作。严格按照法定权限和程序履行职责，切实把行政行为纳入法制化轨道。改革行政执法体制，逐步建立法律顾问制度，完善行政监督机制，强化行政程序建设，提高依法行政能力。加强执法监察，保证政令畅通。深入实施《公务员法》，加强公务员管理，提高公务员法律意识、责任意识和服务意识。

　　努力建设诚信政府。建立健全科学民主决策机制和行政监督机制，完善集体决策、专家咨询和评估、决策听证和公示、决策跟踪反馈和责任追究等各项制度。对重大决策特别是涉及群众切身利益的事项，广泛听取专家和群众意见，提高决策的科学化、民主化水平。建立健全信息公开机制，在政府与公众之间形成良性沟通，增强政府公信力。

　　加强廉政和效能建设。健全教育、制度、监督并重的惩治和预防腐败体系，完善监督和制约机制，规范行政行为。加强行政监察，严格执行廉政建设责任制，认真落实领导干部廉洁自律各项规定，开展专项治理和监督检查，严肃查处违纪违法案件。深入开展效能监察，解决不作为和乱作为等问题。加强节约型政府建设，严格控制办公费用支出。大力提倡重实干、办实事、求实效的工作作风，加强调查研究，坚持采用“课题制”的办法解决重大问题。

　　建设学习、创新型政府。树立现代学习理念，营造讲学习的良好氛围。学习先进地区的新思路和好经验，进一步增强机遇意识、发展意识、创新意识和责任意识，加强管理创新，提高行政能力。加强经济发展、城市建设、社会管理等方面理论和实践的研究，切实增强解决全局性、战略性、关键性问题的本领，全面提高公务员队伍素质。

　　各位代表，今年是哈尔滨解放60周年。回顾过去，令人自豪；展望未来，催人奋进。让我们紧密团结在以胡锦涛同志为总书记的党中央周围，在省委、省政府和市委领导下，紧紧依靠全市人民，振奋精神，同心同德，清正廉洁，真抓实干，为振兴老工业基地，为创造哈尔滨更加美好的未来而努力奋斗！
